# Crystal structure of bis­(1,1,2,2-tetra­methyl­diphosphane-1,2-di­thione-κ^2^ *S*,*S*′)copper(I) tetra­fluorido­borate

**DOI:** 10.1107/S2056989015009913

**Published:** 2015-05-28

**Authors:** Peter W. R. Corfield, Uwe Seeler

**Affiliations:** a Department of Chemistry, Fordham University, 441 East Fordham Road, Bronx, NY 10458, USA; b Department of Chemistry, The Ohio State University, Columbus, Ohio 43210, USA

**Keywords:** crystal structure, diphosphine di­sulfide, copper, C—H. . F hydrogen bonding

## Abstract

In the title compound, [Cu(C_4_H_12_P_2_S_2_)_2_]BF_4_, both diphosphine di­sulfide mol­ecules bind to the Cu^I^ atom, as chelating ligands *via* the S atoms, forming a monovalent cation with a slightly distorted tetra­hedral coordination around the Cu^I^ atom. The average Cu—S distance is 2.350 (15) Å, with small but possibly significant differences within each chelate ring. Ligand P=S distances average 1.964 (3) Å, and the P—P distances are 2.2262 (13) and 2.2166 (14) Å. The ligand chelate rings are twisted in opposite directions, with one in the λ and one in the δ configuration. Although the anisotropic displacement parameters of the F atoms of the anion are quite large compared to that of the B atom, difference Fourier syntheses indicate only one set of sites for the F atoms. In the crystal, possible C—H⋯F hydrogen bonds may stabilize the orientation. The B—F distances, uncorrected for libration, average 1.359 (6) Å.

## Abstract 2

In the title salt, [Cu(C_4_H_12_P_2_S_2_)_2_]BF_4_, both diphosphine di­sulfide mol­ecules bind to the Cu^I^ atom as chelating ligands *via* the S atoms, forming a monovalent cation with a slightly distorted tetra­hedral coordination around the Cu^I^ atom. The ligand chelate rings are twisted in opposite directions, with one in the λ and one in the δ configuration. In the crystal, possible C—H⋯F hydrogen bonds may stabilize the orientation.

## Chemical context   

The title compound was one of a number of phosphine sulfide copper complexes synthesized by Devon Meek and his group (Meek & Nicpon, 1965 ▸). Early reports by Meek and co-workers and by Cotton *et al.* (1974*a* ▸) on coordination complexes of diphosphinedi­sulfide ligands indicated the chelating mode for these ligands to metals such as Cu^I^ as only one of several bonding possibilities, particularly as the chelating model involves rotation about the P—P bond from the *trans* conformation found in the structure of the free ligands (see, for example, Lee & Goodacre, 1971 ▸). Indeed, the tetra­methyl­diphosphinedi­sulfide ligand was shown in one case to bridge copper atoms forming a polymeric chain (Cotton *et al.*, 1974*b* ▸). Our work was initiated to verify the chelating structure that had been predicted for the present compound.
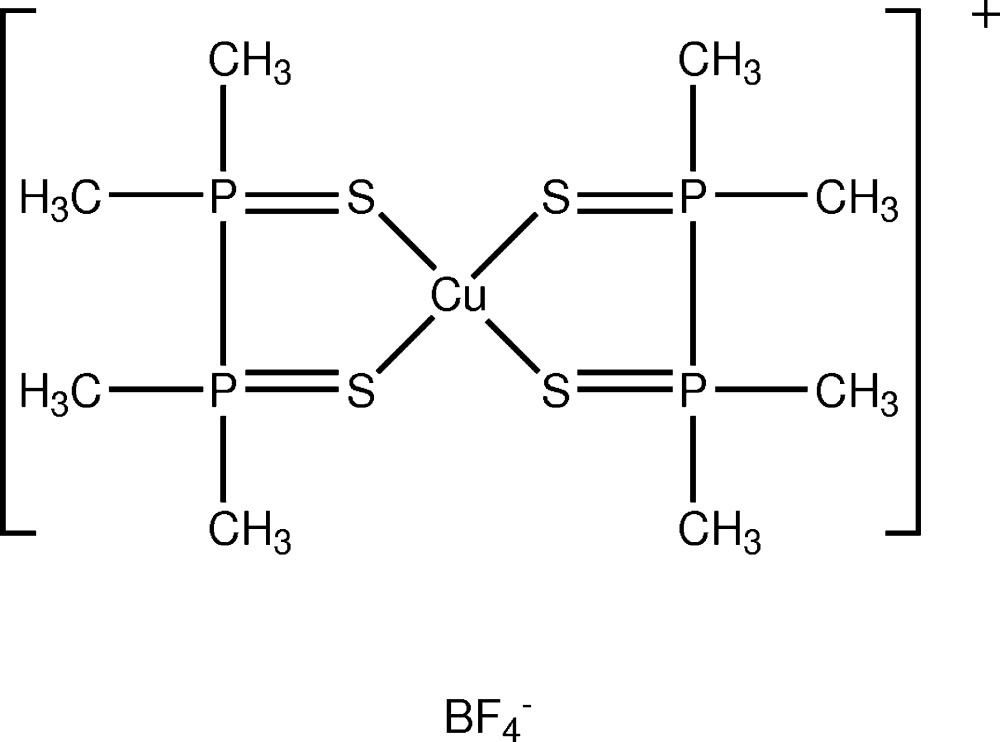


We have reported this structure previously at the 1973 winter meeting of The American Crystallographic Association. The crystal structure of the corresponding hexa­fluorido­phosphate salt has been reported by Liu *et al.* (2003 ▸).

## Structural commentary   

**Figure 1:**
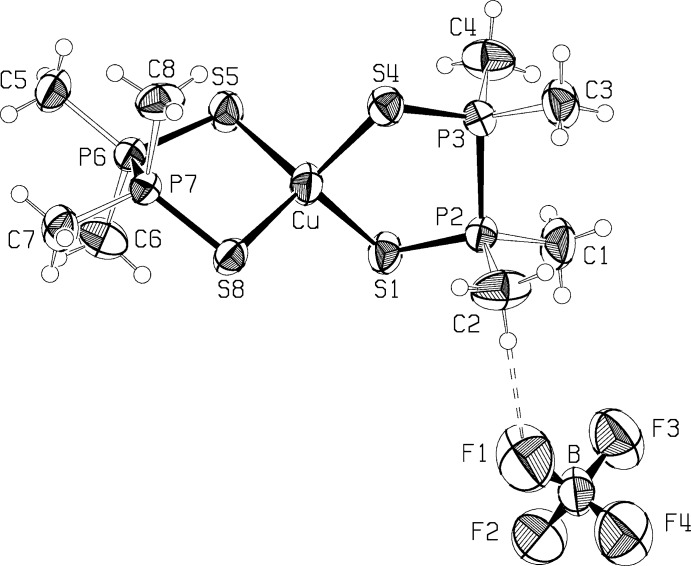
The mol­ecular structure of the title compound, with displacement ellipsoids at the 50% level. The dashed line indicates a hydrogen bond.

In this reported structure, both diphosphine di­sulfide mol­ecules bind to the Cu^I^ atom as chelating ligands *via* the S atoms, forming a monovalent cation with a slightly distorted tetra­hedral coordination around the Cu^I^ (Fig. 1 ▸). Liu *et al.* (2003 ▸) have described the structure of the PF_6_^−^ salt of the present cation, as well as that of the corresponding silver salt.

Selected bond lengths and angles are given in Table 1 ▸. The average Cu—S distance is 2.350 (15) Å, and distances vary by up to 0.065 Å. The chelate S—Cu—S angles are 105.69 (3) and 106.94 (5)°, smaller than the other S—Cu—S angles, which vary from 109.10 (3) to 114.02 (4)° and average 111.1 (10)°. Ligand P=S distances are more constant, with an average of 1.964 (3) Å, and the P—P distances are 2.2262 (13) and 2.2166 (14) Å. The ligand chelate rings are twisted in the λ and δ configurations for S_1_P_2_P_3_S_4_ and S_5_P_6_P_7_S_8_, respectively, with torsional angles about the P—P bonds of 47.97 (6) and −56.37 (6)°. The geometry of the cation, including the slight distortions from regular tetra­hedral geometry at the Cu^I^ atom, is very similar to that seen by Liu *et al.* (2003 ▸).

The BF_4_^−^ anion has regular tetra­hedral geometry, with an average F—B—F angle of 109.5 (6)° and an average B—F distance of 1.359 (6) Å, with distances ranging from 1.347 (5) to 1.370 (5) Å.

## Supra­molecular features   

**Figure 2:**
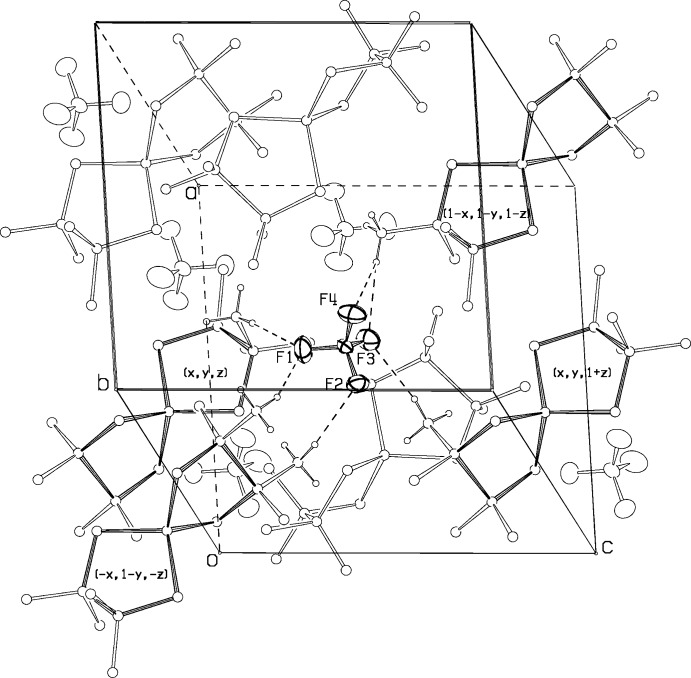
Packing of the title complex, viewed along a direction close to the *b* axis, with ellipsoid outlines for the anion at 30% probability. Putative C—H⋯ F hydrogen bonds from four different cations to the BF_4_^−^ anion are shown.

The packing arrangement in the unit cell is shown in Fig. 2 ▸. There are no unusual features. The shortest inter­molecular contacts not involving F atoms are H4*A*—H8*A*(*x*, 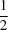 − *y*, 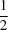 + *z*), at 2.42 Å and H7*B*—H7*C*(−*x*, −*y*, −1 − *z*), at 2.68 Å.

A number of recent structural papers in this journal have postulated that C—H. . . O hydrogen bonds were contributing to packing of organic structures (see, for example: Salas *et al.*, 2011 ▸; Corfield *et al.*, 2014 ▸). This led us to investigate the possibility that F⋯H—C hydrogen bonds were stabilizing the orientation of the BF_4_^−^ ion. We list six putative F⋯H—C hydrogen bonds in Table 2 ▸, and they are represented in Fig. 2 ▸. F⋯C distances are all less than 3.5 Å, and F⋯H distances range from 2.45 to 2.60 Å, while angles at the H atoms are reasonably close to linear.

## Database survey   

A search of the in the Cambridge Structure Database (CSD, Version 5.35; Groom & Allen, 2014 ▸) with a substructure containing the diphosphine di­sulfide ligand of the present study chelated with any metal, *M*, found 11 structures whose coordinates were given. Database P—P and P=S distances average 2.224 (5) and 1.993 (8) Å, while the *M*—S—P and S—P—P angles average 102.1 (9) and 106.1 (6)°, respectively. In the present compound, the P=S distances average 1.965 (2) Å and the average Cu—S—P angle is 98.6 (12)°, both close to values for the other copper(I) compound listed, but somewhat less than values for compounds with other metals. The geometry reflects the lack of π bonding seen in the copper complexes, as indicated by the small change in P=S bond length and ν_P-s_ vibrational mode upon coordination to copper (Liu *et al.*, 2003 ▸). Database torsional angles indicate no preference between λ and δ configurations.

## Synthesis and crystallization   

Details of the synthesis and characterization of a number of phosphine sulfides, including the title compound, are given in Meek & Nicpon (1965 ▸).

## Refinement details   

Crystal data, data collection and structure refinement details are summarized in Table 3 ▸. Each of 18 standard reflections was measured 18–19 times during the 114 h of data collection. No significant crystal decay was noted; indeed we recorded an overall increase in intensity of 1.6% over the entire data collection. No corrections were made. Data were collected in two shells, θ = 0–22.5 and θ = 22.5–35°.

The original data reduction deleted reflections with *I* < 2σ(*I*), and their details are no longer available. Near the end of the final refinements, 2217 missing weak reflections were reinserted into the data file, with *F* ^2^ values set equal to σ(*F* ^2^) found for reflections with F^2^ < 3σ(*F* ^2^), averaged over ten ranges of θ values. The arbitrary assignment of *F* ^2^ values for these weak reflections perhaps explains the high *K* value noted for the weakest reflections in the final refinement, where the *F*_cal_^2^ values will be near zero.

The 6 7 1 reflection was omitted from the final refinements, due to evidence of a transcription error: the chart record clearly indicates a very weak reflection, while the intensity retrieved from our backup storage is very large. Further, the chart record shows that the very strong 1 0 0 reflection was truncated during the scan, and this record was also omitted.

Positions of all non-hydrogen atoms were found by superposition methods. H atoms in the eight methyl groups were constrained to idealized tetra­hedral positions with C—H distances of 0.96 Å. The methyl torsional angles were refined. The *U*_eq_ values for all H atoms were fixed at 1.2 times the *U*_iso_ of their bonded C atoms.

Initial refinements with anisotropic temperature factors for the heavier atoms and constrained hydrogen atom parameters converged smoothly, to *R*_1_ = 0.0443 for 4223 reflections with *F* ^2^ < 2σ. In case there were systematic anisotropic scaling errors in the data collection that might have affected the detailed electron density around the BF_4_^−^ anion, the intensity data were now smoothed by a 12-parameter model with *XABS2* (Parkin *et al.*, 1995 ▸). The smoothing lowered *R*_1_ to 0.0399, but had little effect on the electron density or on the atomic parameters: the average δ/σ was 0.9; two F atoms moved by 3σ.

We made extensive efforts to develop and refine a disordered model for the BF_4_^−^ anion, in light of the large *U_ij_* values for the F atoms, but were unable to find a model with improved *U_ij_* and *R* values. Difference Fourier syntheses phased on the cation parameters always yielded four large peaks corresponding to the current F atom positions; final difference Fourier maps did show several much smaller peaks in the vicinity of the B atom, but no tetra­hedral array emerged.

## Supplementary Material

Crystal structure: contains datablock(s) I. DOI: 10.1107/S2056989015009913/lh5755sup1.cif

Structure factors: contains datablock(s) I. DOI: 10.1107/S2056989015009913/lh5755Isup2.hkl

CCDC reference: 1402327

Additional supporting information:  crystallographic information; 3D view; checkCIF report

## Figures and Tables

**Table 1:** Selected geometric parameters (, )

CuS1	2.3133(15)	CuS4	2.3780(17)
CuS5	2.3719(14)	CuS8	2.3383(13)
			
S1CuS4	105.69(3)	S1CuS5	109.10(3)
S5CuS8	106.94(5)	S4CuS5	110.67(4)
S1CuS8	114.02(4)	S4CuS8	110.46(4)

**Table 2:** Hydrogen-bond geometry (, )

*A*H*D*	*A*H	H*D*	*A* *D*	*A*H*D*
F1H2*C*C2	2.46	0.96	3.397(5)	166.6
F1H5*B*C5^i^	2.57	0.96	3.465(5)	155.8
F2H7*B*C7^i^	2.52	0.96	3.453(4)	163.7
F3H1*C*C1^ii^	2.45	0.96	3.378(5)	163.5
F3H8*B*C8^iii^	2.50	0.96	3.454(5)	170.6
F4H1*C*C1^ii^	2.60	0.96	3.430(5)	144.6

Symmetry codes: (i) *x*, *y*+1, *z*; (ii) *x*+1, *y*+1, *z*+1; (iii) *x*, *y*, *z*+1.

**Table 3:** Experimental details

Crystal data	
Chemical formula	[Cu(C_4_H_12_P_2_S_2_)_2_]BF_4_
*M*_r_	522.74
Crystal system, space group	Monoclinic, *P*2_1_/*c*
Temperature (K)	298
*a*, *b*, *c* ()	12.388(8), 14.903(10), 12.132(7)
()	98.02(2)
*V* (^3^)	2218(2)
*Z*	4
Radiation type	Mo *K*
(mm^1^)	1.68
Crystal size (mm)	0.47 0.29 0.25

Data collection	
Diffractometer	Picker 4-circle
Absorption correction	Gaussian (Busing Levy, 1957 ▸)
*T*_min_, *T*_max_	0.590, 0.691
No. of measured, independent and observed [*I* > 2(*I*)] reflections	6707, 6442, 4223
*R*_int_	0.059
(sin /)_max_ (^1^)	0.703

Refinement	
*R*[*F* ^2^ > 2(*F* ^2^)], *wR*(*F* ^2^), *S*	0.040, 0.102, 1.07
No. of reflections	6442
No. of parameters	207
H-atom treatment	H-atom parameters constrained
_max_, _min_ (e ^3^)	0.41, 0.40

Computer programs: Corfield (1972 ▸, 1973 ▸), *SHELXL97* (Sheldrick, 2008 ▸) and *ORTEPIII* (Burnett Johnson, 1996 ▸).

## supplementary crystallographic information

### Crystal data

**Table:**

[Cu(C_4_H_12_P_2_S_2_)_2_]BF_4_	*F*(000) = 1064
*M**_r_* = 522.74	*D*_x_ = 1.566 Mg m^−^^3^
Monoclinic, *P*2_1_/*c*	Mo *K*α radiation, λ = 0.71070 Å
Hall symbol: -P 2ybc	Cell parameters from 12 reflections
*a* = 12.388 (8) Å	θ = 2.2–29.4°
*b* = 14.903 (10) Å	µ = 1.68 mm^−^^1^
*c* = 12.132 (7) Å	*T* = 298 K
β = 98.02 (2)°	Rod, white
*V* = 2218 (2) Å^3^	0.47 × 0.29 × 0.25 mm
*Z* = 4	

### Data collection

**Table:**

Picker 4-circle diffractometer	4223 reflections with *I* > 2σ(*I*)
Radiation source: sealed X-ray tube	*R*_int_ = 0.059
Oriented graphite 200 reflection monochromator	θ_max_ = 30.0°, θ_min_ = 2.2°
θ/2θ scans	*h* = −17→17
Absorption correction: gaussian (Busing & Levy, 1957)	*k* = 0→20
*T*_min_ = 0.590, *T*_max_ = 0.691	*l* = 0→16
6707 measured reflections	18 standard reflections every 400 reflections
6442 independent reflections	intensity decay: −1.6 (1)

### Refinement

**Table:**

Refinement on *F*^2^	Primary atom site location: heavy-atom method
Least-squares matrix: full	Secondary atom site location: real-space vector search
*R*[*F*^2^ > 2σ(*F*^2^)] = 0.040	Hydrogen site location: inferred from neighbouring sites
*wR*(*F*^2^) = 0.102	H-atom parameters constrained
*S* = 1.07	*w* = 1/[σ^2^(*F*_o_^2^) + (0.*P*)^2^] where *P* = (*F*_o_^2^ + 2*F*_c_^2^)/3
6442 reflections	(Δ/σ)_max_ = 0.002
207 parameters	Δρ_max_ = 0.41 e Å^−^^3^
0 restraints	Δρ_min_ = −0.40 e Å^−^^3^

### Special details

**Table:**

Geometry. All e.s.d.'s (except the e.s.d. in the dihedral angle between two l.s. planes) are estimated using the full covariance matrix. The cell e.s.d.'s are taken into account individually in the estimation of e.s.d.'s in distances, angles and torsion angles; correlations between e.s.d.'s in cell parameters are only used when they are defined by crystal symmetry. An approximate (isotropic) treatment of cell e.s.d.'s is used for estimating e.s.d.'s involving l.s. planes.
Refinement. Refinement of *F*^2^ against ALL reflections. The weighted *R*-factor *wR* and goodness of fit *S* are based on *F*^2^, conventional *R*-factors *R* are based on *F*, with *F* set to zero for negative *F*^2^. The threshold expression of *F*^2^ > σ(*F*^2^) is used only for calculating *R*-factors(gt) *etc*. and is not relevant to the choice of reflections for refinement. *R*-factors based on *F*^2^ are statistically about twice as large as those based on *F*, and *R*-factors based on ALL data will be even larger.

### Fractional atomic coordinates and isotropic or equivalent isotropic displacement parameters (Å^2^)

**Table:**

	*x*	*y*	*z*	*U*_iso_*/*U*_eq_	
Cu	0.21424 (3)	0.38207 (3)	−0.01810 (3)	0.05282 (11)	
S1	0.21067 (6)	0.39393 (6)	0.17147 (6)	0.05262 (19)	
P2	0.36466 (6)	0.41755 (5)	0.22442 (5)	0.04068 (16)	
P3	0.46401 (5)	0.34297 (5)	0.11535 (6)	0.04064 (16)	
S4	0.40133 (6)	0.36787 (5)	−0.03970 (6)	0.05076 (18)	
S5	0.11541 (7)	0.25250 (5)	−0.08606 (6)	0.05343 (19)	
P6	0.00172 (6)	0.31175 (4)	−0.19062 (5)	0.04068 (16)	
P7	0.07818 (6)	0.43287 (4)	−0.25232 (5)	0.03898 (15)	
S8	0.13575 (7)	0.50403 (4)	−0.12096 (6)	0.04904 (17)	
C1	0.4022 (3)	0.3827 (2)	0.3663 (2)	0.0736 (10)	
H1A	0.3808	0.3214	0.3743	0.088*	
H1B	0.3661	0.4202	0.4143	0.088*	
H1C	0.4797	0.3880	0.3862	0.088*	
C2	0.4065 (3)	0.5312 (2)	0.2129 (3)	0.0811 (12)	
H2A	0.3843	0.5520	0.1383	0.097*	
H2B	0.4843	0.5350	0.2301	0.097*	
H2C	0.3733	0.5678	0.2640	0.097*	
C3	0.6038 (2)	0.3765 (2)	0.1469 (3)	0.0691 (9)	
H3A	0.6103	0.4392	0.1310	0.083*	
H3B	0.6473	0.3424	0.1024	0.083*	
H3C	0.6288	0.3658	0.2243	0.083*	
C4	0.4556 (3)	0.2282 (2)	0.1562 (3)	0.0713 (10)	
H4A	0.3818	0.2076	0.1382	0.086*	
H4B	0.4778	0.2230	0.2350	0.086*	
H4C	0.5026	0.1924	0.1175	0.086*	
C5	−0.0506 (3)	0.2437 (2)	−0.3084 (3)	0.0663 (9)	
H5A	0.0082	0.2259	−0.3474	0.080*	
H5B	−0.1030	0.2775	−0.3574	0.080*	
H5C	−0.0850	0.1912	−0.2832	0.080*	
C6	−0.1121 (3)	0.3536 (2)	−0.1301 (3)	0.0658 (9)	
H6A	−0.0866	0.3932	−0.0696	0.079*	
H6B	−0.1508	0.3043	−0.1029	0.079*	
H6C	−0.1601	0.3858	−0.1854	0.079*	
C7	−0.0204 (3)	0.4916 (2)	−0.3488 (2)	0.0595 (8)	
H7A	−0.0773	0.5143	−0.3104	0.071*	
H7B	−0.0510	0.4513	−0.4066	0.071*	
H7C	0.0145	0.5407	−0.3811	0.071*	
C8	0.1809 (3)	0.3893 (2)	−0.3287 (3)	0.0662 (9)	
H8A	0.2259	0.3475	−0.2830	0.079*	
H8B	0.2250	0.4378	−0.3493	0.079*	
H8C	0.1465	0.3595	−0.3945	0.079*	
B	0.2831 (3)	0.6284 (3)	0.5224 (3)	0.0677 (11)	
F1	0.2751 (3)	0.6272 (2)	0.4105 (2)	0.1503 (14)	
F2	0.1801 (2)	0.63147 (17)	0.5507 (2)	0.1085 (8)	
F3	0.3356 (2)	0.55271 (18)	0.5658 (2)	0.1177 (9)	
F4	0.3394 (2)	0.70110 (19)	0.5644 (3)	0.1344 (11)	

### Atomic displacement parameters (Å^2^)

**Table:**

	*U*^11^	*U*^22^	*U*^33^	*U*^12^	*U*^13^	*U*^23^
Cu	0.0528 (2)	0.0585 (2)	0.04281 (19)	0.00125 (17)	−0.00857 (15)	0.00006 (15)
S1	0.0376 (3)	0.0772 (5)	0.0429 (4)	−0.0045 (3)	0.0051 (3)	−0.0063 (3)
P2	0.0381 (3)	0.0449 (4)	0.0380 (3)	−0.0033 (3)	0.0018 (3)	−0.0021 (3)
P3	0.0363 (3)	0.0429 (4)	0.0419 (3)	0.0014 (3)	0.0024 (3)	0.0030 (3)
S4	0.0493 (4)	0.0647 (5)	0.0384 (3)	0.0044 (3)	0.0064 (3)	0.0030 (3)
S5	0.0608 (5)	0.0409 (4)	0.0530 (4)	0.0009 (3)	−0.0116 (3)	0.0046 (3)
P6	0.0428 (4)	0.0377 (3)	0.0396 (3)	0.0002 (3)	−0.0010 (3)	−0.0031 (3)
P7	0.0424 (4)	0.0406 (3)	0.0334 (3)	0.0015 (3)	0.0031 (3)	−0.0025 (3)
S8	0.0629 (4)	0.0370 (3)	0.0429 (3)	0.0003 (3)	−0.0076 (3)	−0.0045 (3)
C1	0.069 (2)	0.110 (3)	0.0381 (15)	0.008 (2)	−0.0074 (15)	0.0023 (17)
C2	0.080 (3)	0.0507 (19)	0.121 (3)	−0.0200 (18)	0.045 (2)	−0.022 (2)
C3	0.0392 (16)	0.096 (3)	0.071 (2)	−0.0017 (17)	0.0006 (15)	−0.0036 (19)
C4	0.095 (3)	0.0497 (18)	0.072 (2)	0.0151 (18)	0.023 (2)	0.0154 (16)
C5	0.074 (2)	0.0553 (18)	0.0617 (19)	−0.0028 (16)	−0.0176 (17)	−0.0142 (15)
C6	0.060 (2)	0.063 (2)	0.078 (2)	0.0063 (16)	0.0247 (17)	0.0072 (17)
C7	0.067 (2)	0.067 (2)	0.0403 (14)	0.0046 (16)	−0.0050 (13)	0.0113 (14)
C8	0.067 (2)	0.069 (2)	0.067 (2)	0.0032 (17)	0.0263 (18)	−0.0135 (16)
B	0.052 (2)	0.085 (3)	0.062 (2)	0.000 (2)	−0.0063 (18)	0.000 (2)
F1	0.134 (3)	0.248 (4)	0.0717 (17)	0.049 (2)	0.0223 (17)	0.0095 (19)
F2	0.0738 (15)	0.140 (2)	0.116 (2)	−0.0084 (15)	0.0288 (14)	−0.0204 (16)
F3	0.102 (2)	0.0990 (19)	0.145 (2)	0.0125 (16)	−0.0075 (17)	0.0219 (18)
F4	0.103 (2)	0.099 (2)	0.192 (3)	−0.0228 (17)	−0.010 (2)	−0.009 (2)

### Geometric parameters (Å, º)

**Table:**

Cu—S1	2.3133 (15)	C2—H2C	0.9600
Cu—S5	2.3719 (14)	C3—H3A	0.9600
Cu—S4	2.3780 (17)	C3—H3B	0.9600
Cu—S8	2.3383 (13)	C3—H3C	0.9600
S1—P2	1.9580 (15)	C4—H4A	0.9600
P2—C2	1.782 (3)	C4—H4B	0.9600
P2—C1	1.796 (3)	C4—H4C	0.9600
P2—P3	2.2262 (13)	C5—H5A	0.9600
P3—C4	1.788 (3)	C5—H5B	0.9600
P3—C3	1.792 (3)	C5—H5C	0.9600
P3—S4	1.9677 (14)	C6—H6A	0.9600
S5—P6	1.9683 (13)	C6—H6B	0.9600
P6—C6	1.791 (3)	C6—H6C	0.9600
P6—C5	1.798 (3)	C7—H7A	0.9600
P6—P7	2.2166 (14)	C7—H7B	0.9600
P7—C8	1.796 (3)	C7—H7C	0.9600
P7—C7	1.797 (3)	C8—H8A	0.9600
P7—S8	1.9637 (12)	C8—H8B	0.9600
C1—H1A	0.9600	C8—H8C	0.9600
C1—H1B	0.9600	B—F4	1.349 (5)
C1—H1C	0.9600	B—F2	1.369 (5)
C2—H2A	0.9600	B—F3	1.370 (5)
C2—H2B	0.9600	B—F1	1.347 (5)
			
S1—Cu—S4	105.69 (3)	H2A—C2—H2C	109.5
S5—Cu—S8	106.94 (5)	H2B—C2—H2C	109.5
S1—Cu—S8	114.02 (4)	P3—C3—H3A	109.5
S1—Cu—S5	109.10 (3)	P3—C3—H3B	109.5
S4—Cu—S5	110.67 (4)	H3A—C3—H3B	109.5
S4—Cu—S8	110.46 (4)	P3—C3—H3C	109.5
Cu—S1—P2	100.78 (4)	H3A—C3—H3C	109.5
C1—P2—C2	108.11 (18)	H3B—C3—H3C	109.5
C1—P2—S1	111.87 (13)	P3—C4—H4A	109.5
C2—P2—S1	115.18 (14)	P3—C4—H4B	109.5
C1—P2—P3	109.56 (13)	H4A—C4—H4B	109.5
C2—P2—P3	103.71 (12)	P3—C4—H4C	109.5
S1—P2—P3	108.02 (5)	H4A—C4—H4C	109.5
C4—P3—C3	107.42 (17)	H4B—C4—H4C	109.5
C4—P3—S4	114.52 (12)	P6—C5—H5A	109.5
C3—P3—S4	113.12 (12)	P6—C5—H5B	109.5
C4—P3—P2	104.74 (12)	H5A—C5—H5B	109.5
C3—P3—P2	109.37 (12)	P6—C5—H5C	109.5
S4—P3—P2	107.27 (5)	H5A—C5—H5C	109.5
P3—S4—Cu	99.91 (5)	H5B—C5—H5C	109.5
Cu—S5—P6	98.45 (5)	P6—C6—H6A	109.5
C5—P6—C6	107.78 (17)	P6—C6—H6B	109.5
C5—P6—S5	113.90 (12)	H6A—C6—H6B	109.5
C6—P6—S5	115.20 (13)	P6—C6—H6C	109.5
C5—P6—P7	108.35 (12)	H6A—C6—H6C	109.5
C6—P6—P7	104.64 (12)	H6B—C6—H6C	109.5
S5—P6—P7	106.38 (6)	P7—C7—H7A	109.5
C8—P7—C7	107.79 (16)	P7—C7—H7B	109.5
C8—P7—S8	114.24 (13)	H7A—C7—H7B	109.5
C7—P7—S8	113.73 (11)	P7—C7—H7C	109.5
C8—P7—P6	104.29 (12)	H7A—C7—H7C	109.5
C7—P7—P6	109.48 (12)	H7B—C7—H7C	109.5
S8—P7—P6	106.83 (5)	P7—C8—H8A	109.5
P7—S8—Cu	95.17 (6)	P7—C8—H8B	109.5
P2—C1—H1A	109.5	H8A—C8—H8B	109.5
P2—C1—H1B	109.5	P7—C8—H8C	109.5
H1A—C1—H1B	109.5	H8A—C8—H8C	109.5
P2—C1—H1C	109.5	H8B—C8—H8C	109.5
H1A—C1—H1C	109.5	F1—B—F2	108.2 (3)
H1B—C1—H1C	109.5	F1—B—F3	109.9 (4)
P2—C2—H2A	109.5	F1—B—F4	110.6 (4)
P2—C2—H2B	109.5	F2—B—F3	109.9 (4)
H2A—C2—H2B	109.5	F2—B—F4	109.2 (4)
P2—C2—H2C	109.5	F3—B—F4	108.9 (3)
			
Cu—S1—P2—P3	−33.85 (5)	Cu—S5—P6—P7	31.27 (5)
S1—P2—P3—S4	47.97 (6)	S5—P6—P7—S8	−56.37 (6)
P2—P3—S4—Cu	−32.97 (5)	P6—P7—S8—Cu	45.02 (5)

### Hydrogen-bond geometry (Å, º)

**Table:**

*D*—H···*A*	*D*—H	H···*A*	*D*···*A*	*D*—H···*A*
C1—H1*C*···F4^i^	0.96	2.60	3.430 (5)	145
C1—H1*C*···F3^i^	0.96	2.45	3.378 (5)	164
C2—H2*C*···F1	0.96	2.46	3.397 (5)	167
C5—H5*B*···F1^ii^	0.96	2.57	3.465 (5)	156
C7—H7*B*···F2^ii^	0.96	2.52	3.453 (4)	164
C8—H8*B*···F3^iii^	0.96	2.50	3.454 (5)	171

Symmetry codes: (i) −*x*+1, −*y*+1, −*z*+1; (ii) −*x*, −*y*+1, −*z*; (iii) *x*, *y*, *z*−1.
